# Evaluation of the prevalence of asthma and chronic obstructive pulmonary disease among opium users, and cigarette smokers and comparison with normal population in Kharameh: a cross-sectional study

**DOI:** 10.1186/s12890-023-02734-8

**Published:** 2023-11-01

**Authors:** Laleh Raeisy, Seyed Masoom Masoompour, Abbas Rezaianzadeh

**Affiliations:** 1 Student Research committee of medical school, Shiraz University of Medical Sciences, Shiraz, Iran; 2 Non-Communicable Diseases Research Center, Shiraz University of Medical Sciences, Shiraz, Iran; 3 Colorectal Research Center, Shiraz University of Medical Sciences, Shiraz, Iran

**Keywords:** Cohort, COPD, Asthma, Prevalence, Iran

## Abstract

**Background:**

Recent studies have suggested that opium use may increase mortality from pulmonary diseases. However, there are limited comprehensive studies regarding the prevalence of Asthma and Chronic Obstructive Pulmonary Disease (COPD) among tobacco and opium users has been published. We aimed to determine the prevalence of respiratory disease among tobacco and opium users.

**Methods:**

This cross-sectional study of tobacco and opium users and matched controls was conducted in the Kharameh Cohort, Fars, Iran. The prevalence of COPD and asthma, along with the participants demographical and spirometry data were examined.

**Results:**

The average age of participants was 57 ± 8 years. Never smokers had a significant higher BMI (26.6 vs. 24.8), FEV1 (91% vs. 82%) and FVC (96% vs. 88%) values compared to participants with a positive smoking status. There was a statistical difference in the prevalence of COPD, asthma, and asthma COPD overlap (ACO) based on the participants smoking status, with the highest prevalence among opium and cigarette smokers, followed by opium users alone. Based on multivariate analysis, higher age, lower BMI, lower education than under diploma, cigarette smoking and opium use were significantly correlated with higher COPD prevalence; while lower age, cigarette smoking and opium use were significantly correlated with higher asthma prevalence. Illiterate participants had a significantly higher prevalence of COPD (23.6%), asthma (22%), and ACO (7.9%) among the educational groups. Regarding the prevalence of asthma, the higher socio-economic group had the lowest prevalence.

**Conclusions:**

Opium and tobacco users had a significantly higher prevalence of respiratory diseases, along with lower lung function tests based on spirometry evaluation.

## Background

Cigarette smoking has been shown to worsen the lung function [1], increase exacerbation rates, [2, 3] and increase respiratory symptoms [4, 5] in people with chronic obstructive pulmonary disease (COPD) or asthma [6]. Furthermore, when compared to the risk associated with smoking in other health conditions, smoking confers a relative risk of mortality that is two times higher in COPD [7]. Despite the significant negative health effects of smoking in COPD and asthma, precise estimates of smoking prevalence in COPD and asthma compared to the general smoking population, as well as analyses of key variables that may underpin this (such as sociodemographic characteristics, smoking behaviors, and use of physician counseling and smoking cessation pharmacotherapy), are lacking. Understanding the scope and nature of smoking behaviors and cessation strategies in COPD and asthma may help healthcare providers and policymakers’ better target resources and develop more effective approaches to assist smokers in these high-risk disease groups in successfully quitting. Despite the fact that smoking-related lung diseases such as COPD and asthma are on the rise, the effects of demographical factors on smoking behavior and cessation among those with respiratory diseases are poorly understood [8–11].

Substance abuse is defined as the harmful use of any substance that has not been approved by medical authorities and are being used for recreational purposes worldwide [12]. Among the members of substance abuse families is opium, which is commonly used to relieve pain in middle-aged people. It’s a substance derived from the poppy plant. Its history can be traced all the way back to antiquity. Opium was used in traditional medicine in Iran, where it was known as Afion or Taryak [13]. Opium can be consumed as an inhalant or as a comestible. Opioid users have been reported to have small airway diseases. In 1976, the first study of the link between opium and COPD was published in Tehran, Iran [14]. Lung diseases with an obstructive pattern can be clinically demonstrated in opium smokers [15]. Chronic bronchitis, emphysema, bronchiolitis, and COPD can all be caused by opium use [14, 15]. The impact of opium on the respiratory system has not been thoroughly studied; however, according to evidence, opium inhalation can be classified as a risk factor in COPD epidemiology [15].

Asthma and COPD [15] have similarities in that both can present with fixed airway obstruction (6). Asthma, on the other hand, has been identified as a risk factor for the development of COPD [16]. Bronchial hyper-responsiveness (BHR) is a defining feature of asthma, and it can be found in 54% of COPD patients [17]. Asthma and COPD patients should avoid opiates due to the increased mortality rate [18].

The majority of respiratory guidelines agree that spirometry should be used to diagnose COPD [19]. Patients with respiratory complaints in most countries, on the other hand, go to their primary care provider first, rather than a pulmonary specialist [20, 21], because access to spirometry is limited by cost, availability of spirometry equipment, availability of appropriately trained personnel, or simply the time required for the test. Based on available data, spirometry appears to be underutilized in primary care [22, 23].

The goal of this study is to compare the prevalence of asthma and COPD among opium, and cigarette smokers with COPD or asthma to the general population, and to see if smoking habits, physician counseling about quitting smoking, and smoking cessation medication use differed between smokers with COPD or asthma and the general smoking population. We also aim to evaluate the prevalence of COPD and related risk factors.

## Methods

### Study design and participants

This analytical cross-sectional study was conducted from March to July 2022 using the baseline data of the Kharameh cohort study, a branch of the Prospective Epidemiological Research Studies in Iran (PERSIAN cohort). The rationale, objectives, and design of the PERSIAN cohort study have earlier been published [24, 25]. Kharameh is one of the counties of Fars province, the fifth populous province located in southwestern Iran. According to the latest national census in 2016, its population was 54,864 [26]. Out of the participants in the Kharameh cohort aged 45 to 75 years old, 5944 were female and 4719 were male. The inclusion criteria were living in Kharameh for at least 1 year before the start of this study, and willingness to participate. The participants were categorized into three groups of opium user, cigarette smokers, and a combination of both. Because of very low prevalence of opium among women, they were excluded in this study.

Using statulator, online sample size calculator, With confidence of 95% and absolute precision of 5%, based on previous study on the prevalence of COPD and asthma in patients with opioid dependency [27], the sample size of our study was estimated to be 350 [28]. Patients in each group were selected by systematic random sampling method, and were matched based on their age.

### Substance and opium usage

Opium details included types of opiates commonly used, such as teryak, heroin, sukhteh, and shireh, age of onset and duration of use, opium administration routes, typical amount of use in nokhods (the local unit for opium use, equivalent to 0.195 g), and frequency of use were all included (how many days in a week if weekly or more). Participants were also questioned if they had ever used opium as during their earlier periods in their lifetime [29].

Cigarette smokers were defined as those who had used cigarettes for at least six months, otherwise classified as never smoker. The total amount of cigarettes consumed was calculated in pack-years [30].

### Disease diagnosis

Asthma and COPD diagnosis were based on the patients history and spirometry results, and also the Global Initiative for Asthma and Global Initiative for Chronic Obstructive Lung Disease criteria for diagnosis of diseases of chronic airflow limitation: asthma, COPD and asthma-COPD overlap syndrome (ACOS) [31]. Patients’ symptoms and history in favor of asthma include cough, wheezing, symptoms during nighttime, exaggeration of symptoms during physical activity or excitement, with variability of symptoms throughout different periods. Features in favor of COPD include productive cough, progressive increase in severity of accompanying symptoms. For spirometry evaluation, for asthma we included variable airflow limitation, along with the increase of FEV1 of more than 12% and more then 200mL from baseline after 2 puffs (each puff 100 µg) of salbutamol spray as bronchodilator (BD). For COPD, persistent airflow limitation along with post BD FEV1/FVC ratio of less than 0.7 was considered. For ACOS, FEV1 higher than 12% and above 300mL from the baseline after BD, but FEV1 to FVC ratio of under 0.7 after BD. Also, a normal FEV1 to FVC ratio before and after BD is in favor of asthma and contrary to COPD and ACOS [31].

### Data Collection

Data were collected with a checklist consisting of three main parts: socio-demographic characteristics, lifestyle variables, and anthropometric indices (height and weight). Socio-demographic variables are comprised of age, marital status (married, single, widowed/divorced), educational level (illiterate, under diploma, high school diploma, university degree), occupational status (employed, unemployed), and SES (low, intermediate, and high). The socio-economic status (SES) of households was calculated using the principal component analysis method and included assets of the participants such as type of residence (owned or rented), residential area (in square meters), number of rooms, ownership of landline telephone, washing machine, dishwasher, flat-screen TV, refrigerator, vacuum cleaner, or personal computer/laptop, access to Internet at home, access to a shower and toilet, and car ownership status and its value.

Lifestyle variables included history of cigarette smoking (yes-no), amount and years of smoking, type and frequency of opium usage.

Weight and height of participants were measured and BMI was calculated accordingly.

Based on American thoracic society recommendations, spirometry evaluation was performed by a trained professional and the FloXpert spirometry device, in which after measuring the FEV1 and FVC, the patient is administered 2 puffs (each puff 100 µg) of salbutamol (Iran Daro Co.), and re-evaluated with spirometry after 20 min.

### Data analysis

The Statistical Package for Social Sciences (SPSS) software version 21 (IBM, Armonk, NY, USA) was used for data analysis. Descriptive analysis including mean, standard deviation, and frequency distribution was done to assess demographic and anthropometric characteristics. Chi-square test was used for associations between the independent variables and the outcome variable. A binary logistic regression analysis was applied to determine the predictive variables of COPD and asthma among adults in Kharameh. According to the univariate analyses, variables with *p* ≤ 0.2 had the necessary requirement to be entered into the regression model. The final model is reported with odds ratios (ORs) and 95% confidence intervals (CIs). A *p*-value less than 0.05 was considered significant.

## Results

Among the participants, the average age was 57 ± 8 years, with an average BMI of 25.7 ± 4.22 kg/m^2^. The majority of participants had an educational level of under diploma (41%), was married (98%), and was employed (82.9%), and had an intermediate social status (56%). Table 1 demonstrates the baseline features of our population.

**Table 1:** Baseline demographical and social features of a cohort population in Kharameh, Fars

Variable		Value
Age (years); **mean ± SD**		57 ± 8
Height (cm); **mean ± SD**		171 ± 6
Weight (kg); **mean ± SD**		77 ± 37
Body mass index (kg/m^2^); **mean ± SD**		25.7 ± 4.22
Education; **n (%)**	Illiterate	127 (36.7%)
	Under diploma	142 (41.0%)
	Diploma	44 (12.7%)
	Academic	33 (9.5%)
Marital status; **n (%)**	married	339 (98.0%)
	single	4 (1.2%)
	widow	2 (0.6%)
	divorced	1 (0.3%)
Occupation; **n (%)**	Employed	287 (82.9%)
	jobless	59 (17.1%)
Socio-economic status; **n (%)**	low	112 (32.4%)
	intermediate	192 (55.5%)
	high	42 (12.1%)
Smoking status; **n (%)**	Never never smoker	185 (53.5%)
	opium user	90 (26.0%)
	cigarette smoker	61 (17.6%)
	opium and cigarette	10 (2.9%)

We evaluated the patients features based on their occupation, in which the results are demonstrated in Table 2. As demonstrated, employed participants had a significant lower age among the demographic features. There was no statistically significant difference in the spirometry results or the prevalence of COPD, asthma, and ACO based on the participants occupations.

**Table 2:** Evaluation of the demographical and spirometry features of a cohort population in Kharameh, Fars based on the participants occupation

Value		Occupation		P-Value
		Employed	Unemployed	
Age (years); **mean ± SD**		57 ± 7	62 ± 8	< 0.001
Height (cm); **mean ± SD**		171 ± 6	171 ± 7	0.682
Weight (kg); **mean ± SD**		77 ± 40	75 ± 16	0.728
Body mass index (kg/m^2^); **mean ± SD**		25.69 ± 4.12	25.72 ± 4.67	0.972
FEV1%; mean ± SD	Pre-Bronchodilator	85 ± 16	82 ± 21	0.179
	Post-Bronchodilator	94 ± 15	91 ± 19	0.169
FVC%; mean ± SD	Pre-Bronchodilator	89 ± 16	86 ± 21	0.159
	Post-Bronchodilator	95 ± 15	92 ± 21	0.172
COPD; n (%)	No	257 (89.5%)	48 (81.4%)	0.065
	Yes	30 (10.5%)	11 (18.6%)	
Asthma; n (%)	No	239 (83.3%)	51 (86.4%)	0.351
	Yes	48 (16.7%)	8 (13.6%)	
ACO; n (%)	No	278 (96.9%)	57 (96.6%)	0.587
	Yes	9 (3.1%)	2 (3.4%)	

We evaluated the patients features based on their smoking, in which the results are demonstrated in Table 3. As demonstrated, never smoker participants had a significant higher BMI among the demographic features. Also, never smokers had a significantly higher FEV1 and FVC values compared to participants with a positive smoking status. However, there was a statistical difference in the prevalence of COPD, asthma, and ACO based on the participants smoking status, with the highest prevalence among opium and cigarette smokers, followed by opium users alone.

**Table 3:** Evaluation of the demographical and spirometry features of a cohort population in Kharameh, Fars based on the participants smoking status

Value		Smoking Status				P-Value
		Never smoker	Opium User	Cigarette Smoker	Opium And Cigarette	
Age (years); **mean ± SD**		57 ± 8	59 ± 9	57 ± 7	59 ± 5	0.156
Height (cm); **mean ± SD**		171 ± 6	171 ± 6	172 ± 6	168 ± 6	0.342
Weight (kg); **mean ± SD**		81 ± 48	72 ± 14	74 ± 14	66 ± 12	0.149
Body mass index (kg/m^2^); **mean ± SD**		26.62 ± 3.98	24.64 ± 4.17	24.86 ± 4.43	23.20 ± 3.86	**0.001**
FEV1% Mean ± SD	Pre-Bronchodilator	88 ± 15	82 ± 18	79 ± 17	82 ± 14	**0.001**
	Post-Bronchodilator	91 ± 15	86 ± 18	82 ± 17	95 ± 20	**0.001**
FVC%; Mean ± SD	Pre-Bronchodilator	95 ± 15	93 ± 17	87 ± 15	97 ± 11	**0.013**
	Post-Bronchodilator	96 ± 16	95 ± 15	88 ± 17	105 ± 20	**0.002**
COPD; N (%)	No	184 (99.5%)	65 (72.2%)	50 (82.0%)	6 (60.0%)	**< 0.001**
	Yes	1 (0.5%)	25 (27.8%)	11 (18.0%)	4 (40.0%)	
Asthma; N (%)	No	177 (95.7%)	59 (65.6%)	48 (78.7%)	6 (60.0%)	**< 0.001**
	Yes	8 (4.3%)	31 (34.4%)	13 (21.3%)	4 (40.0%)	
ACO; N (%)	No	184 (99.5%)	85 (94.4%)	58 (95.1%)	8 (80.0%)	**0.001**
	Yes	1 (0.5%)	5 (5.6%)	3 (4.9%)	2 (20.0%)	

We evaluated the patients features based on their education level, in which the results are demonstrated in Table 4. As demonstrated, illiterate participants had a significant higher age and also lower height among the demographic features, however, there was no significant difference based on the BMI levels. Based on spirometry results, participants with an academic level of education had a significantly higher pre-bronchodilator and post-bronchodilator FEV1 and also higher pre-bronchodilator FVC values compared to the other educational groups. There was a statistically significant difference in the prevalence of COPD, asthma, and ACO based on the participants education status, with illiterate participants having the highest prevalence among the educational groups.

**Table 4:** Evaluation of the demographical and spirometry features of a cohort population in Kharameh, Fars based on the participants education status

Value		Education Status				P-Value
		Illiterate	Under Diploma	Diploma	Academic	
Age (years); **mean ± SD**		61 ± 8	56 ± 7	53 ± 6	54 ± 6	**< 0.001**
Height (cm); **mean ± SD**		170 ± 7	172 ± 6	173 ± 6	171 ± 6	**0.027**
Weight (kg); **mean ± SD**		73 ± 14	80 ± 55	79 ± 17	77 ± 13	0.381
Body mass index (kg/m^2^); **mean ± SD**		25.09 ± 4.33	25.76 ± 4.30	26.86 ± 3.72	26. 23 ± 33.72	0.087
FEV1% Mean ± SD	Pre-Bronchodilator	81 ± 19	87 ± 14	84 ± 16	91 ± 17	**0.004**
	Post-Bronchodilator	91 ± 17	95 ± 14	91 ± 17	100 ± 16	**0.010**
FVC%; Mean ± SD	Pre-Bronchodilator	85 ± 18	90 ± 16	88 ± 15	94 ± 18	**0.016**
	Post-Bronchodilator	93 ± 16	95 ± 16	93 ± 16	98 ± 22	0.420
COPD; N (%)	No	97 (76.4%)	135 (95.1%)	43 (97.7%)	30 (90.9%)	**< 0.001**
	Yes	30 (23.6%)	7 (4.9%)	1 (2.3%)	3 (9.1%)	
Asthma; N (%)	No	99 (78.0%)	119 (83.8%)	41 (93.2%)	31 (93.9%)	**0.036**
	Yes	28 (22.0%)	23 (16.2%)	3 (6.8%)	2 (6.1%)	
ACO; N (%)	No	117 (92.1%)	142 (100.0%)	43 (97.7%)	33 (100%)	**0.002**
	Yes	10 (7.9%)	0 (0.0%)	1 (2.3%)	0 (0%)	

We then evaluated the patients features based on their SES status, in which the results are demonstrated in Table 5. As demonstrated, low SES participants had a significant highest age and also the lowest height and BMI among the demographic features. Based on spirometry results, participants with a low SES level had a significantly lower pre-bronchodilator FEV and FVC results, while the intermediate SES group had the highest pre-bronchodilator FEV and FVC results. Regarding the prevalence of asthma, the higher SES group had the lowest prevalence while the low SES group had the highest prevalence of asthma. There was no significant difference among the SES group regarding the usage of opium and cigarettes.

**Table 5:** Evaluation of the demographical and spirometry features of a cohort population in Kharameh, Fars based on the participants socio-economic status

Value		Socio-economic Status			P-Value
		Low	Intermediate	High	
Age (years); mean ± SD		59 ± 8	56 ± 7	58 ± 6	**0.013**
Height (cm); mean ± SD		169 ± 6	172 ± 6	172 ± 6	**0.002**
Weight (kg); mean ± SD		70 ± 15	80 ± 47	79 ± 12	0.056
Body mass index (kg/m^2^); mean ± SD		24.68 ± 4.30	26.05 ± 4.25	26.79 ± 3.26	**0.005**
FEV1% mean ± SD	Pre-Bronchodilator	80 ± 19	87 ± 15	85 ± 13	**0.003**
	Post-Bronchodilator	91 ± 17	95 ± 15	92 ± 16	0.135
FVC%; mean ± SD	Pre-Bronchodilator	84 ± 18	91 ± 16	87 ± 14	**0.004**
	Post-Bronchodilator	93 ± 16	96 ± 17	93 ± 14	0.438
COPD; n (%)	No	93 (83.0%)	175 (91.1%)	37 (88.1%)	0.108
	Yes	19 (17.0%)	17 (8.9%)	5 (11.9%)	
Asthma; n (%)	No	85 (75.9%)	165 (85.9%)	40 (95.2%)	**0.007**
	Yes	27 (24.1%)	27 (14.1%)	2 (4.8%)	
ACO; n (%)	No	106 (94.6%)	187 (97.4%)	42 (100.0%)	0.191
	Yes	6 (5.4%)	5 (2.6%)	0 (0.0%)	
Smoking Status; n (%)	Never smoker	54 (29.2%)	107 (57.8%)	24 (13.0%)	0.056
	Opium User	36 (40.0%)	49 (54.4%)	5 (5.6%)	
	Cigarette Smoker	19 (31.1%)	29 (47.5%)	13 (21.3%)	
	Opium And Cigarette	3 (30.0%)	7 (70.0%)	0 (0.0%)	

Table 6 demonstrated the odd’s ratio of variables in COPD and Asthma. As demonstrated, higher age, lower BMI, lower education than under diploma, cigarette smoking and opium use were significantly correlated with higher COPD prevalence; while lower age, cigarette smoking and opium use were significantly correlated with higher asthma prevalence.

**Table 6:** The Odd’s ratio of variables in COPD and Asthma

Variables	Subclass	COPD				Asthma			
		Multivariate		Univariate		Multivariate		Univariate	
		Odd’s ratio (95% CI)	P value	Odd’s ratio (95% CI)	P value	Odd’s ratio (95% CI)	P value	Odd’s ratio (95% CI)	P value
**Age (yr)**		1.13 (1.05_1.21)	**0.001**	1.11 (1.06_1.16)	**< 0.001**	0.94 (0.9_0.99)	**0.019**	0.98 (0.95_1.02)	0.349
**BMI (Kg/m2)**		0.7 (0.6_0.81)	**< 0.001**	0.73 (0.66_0.81)	**< 0.001**	1.01 (0.93_1.09)	0.857	0.94 (0.87_1)	0.059
**Education**	*Illiterate*	Baseline							
	*Under diploma*	0.17 (0.06_0.53)	**0.002**	0.17 (0.07_0.4)	**< 0.001**	0.78 (0.38_1.63)	0.511	0.68 (0.37_1.26)	0.223
	*Diploma*	0.16 (0.01_2.73)	0.205	0.08 (0.01_0.57)	**0.012**	0.34 (0.09_1.36)	0.127	0.26 (0.07_0.9)	**0.033**
	*Academic*	1.08 (0.14_8.62)	0.941	0.32 (0.09_1.14)	0.078	0.36 (0.07_1.96)	0.236	0.23 (0.05_1.01)	0.052
**Occupation**		1.98 (0.6_6.59)	0.266	1.96 (0.92_4.18)	0.081	1.18 (0.46_3.03)	0.737	0.78 (0.35_1.75)	0.548
**Socio-economic Status**	*Low*	Baseline							
	*Intermediate*	0.81 (0.3_2.21)	0.683	0.48 (0.24_0.96)	**0.038**	0.54 (0.27_1.09)	0.085	0.52 (0.28_0.93)	0.029
	*High*	4.24 (0.82_22.05)	0.086	0.66 (0.23_1.9)	0.443	0.27 (0.06_1.27)	0.097	0.16 (0.04_0.7)	**0.015**
**Cigarette Smoking**		9.25 (2.78_30.78)	**< 0.001**	2.57 (1.28_5.16)	**0.008**	3.61 (1.62_8.03)	**< 0.001**	1.91 (1_3.62)	**0.049**
**Opium user**		21.12 (6.37_69.95)	**< 0.001**	7.97 (3.86_16.42)	**< 0.001**	7.84 (3.8_16.2)	**< 0.001**	5.77 (3.14_10.59)	**< 0.001**

## Discussion

We evaluated the prevalence of asthma and COPD among men in Kharameh, Fars, to assess whether opium and smoking can affect the incidence of asthma and COPD. Among the participants, the average age was 57 ± 8 years, with an average BMI of 25.7 ± 4.22 kg/m^2^. Based on our demographical results, never smoker participants had a significant higher BMI among the demographic features, and low SES participants had a significant higher age and also the lowest height and BMI among the demographic features. There was statistically significant difference regarding the prevalence of COPD, asthma, and ACO based on the participants occupations and smoking status. However, there was a statistically significant difference in the prevalence of COPD, asthma, and ACO based on the participants education and SES status, with illiterate participants having the highest prevalence among the educational groups, and higher SES group had the lowest prevalence while the low SES group had the highest prevalence of asthma based on SES status. Therefore, based on our findings, opium and smoking had a significant relation to the prevalence of asthma and COPD in our cohort. higher age, lower BMI, lower education than under diploma, cigarette smoking and opium use were significantly correlated with higher COPD prevalence; while lower age, cigarette smoking and opium use were significantly correlated with higher asthma prevalence.

Previous studies have reported various correlations between pulmonary diseases and opium use [32–36]. Similar to our results, Palmer et al. demonstrated a relation between drug misuse and receiving a diagnosis of COPD or asthma and respiratory prescriptions [37]. Furthermore, Levine et al. [38] reported a connection between cocaine abuse and admissions to intensive care units as well as a link between heroin or cocaine abuse and higher intubation rates in asthma flare-ups.

Buster et al. [39], Lewis-Burke et al. [40], and Mitchell et al. [41] studied and described pulmonary morbidity in opioid-dependent patients. COPD in people with opioid use disorder (POUD) is often diagnosed late, when lung function and life expectancy have already been reduced, or not at all, as it is in the general population [42]. Furthermore, investigating COPD prevalence among opioid agonist treatment patients is an important first step in ensuring and improving timely medical treatment offers for the opioid agonist treatment cohort and avoiding costs (e.g. fewer hospitalizations due to exacerbations).

Based on spirometry evolution, never smokers had a significantly higher FEV1 and FVC values compared to participants with a positive smoking status. Participants with an academic level of education had a significantly higher pre-bronchodilator and post-bronchodilator FEV1 and also higher pre-bronchodilator FVC values compared to the other educational groups. Participants with a low SES level had a significantly lower pre-bronchodilator FEV1 and FVC results, while the intermediate SES group had the highest pre-bronchodilator FEV and FVC results. There was no statistical difference in the spirometry results based on the participants’ occupations and smoking status. Aldington et al. also found that patients who used both tobacco and cannabis had lower FEV1, despite the fact that cannabis usage alone had no effect on FEV1 (when compared to never smokers) [43]. Little cannabis usage has no impact on pulmonary function, according to a study by Pletcher et al. [43].

There was no statistically significant difference among the SES group regarding the usage of opium and cigarettes. Although our findings demonstrated a higher prevalence of pulmonary diseases among opium and cigarette users, the high rate of tobacco use among drug users cannot entirely account for the link between opium use and chronic respiratory disease. This implies that there might be more intricate elements relating to opium abuse that require more investigation. The connections may result from societal factors, the route of drug abuse, or the pharmacological effects of illicit drugs on the airways. Medical personnel that interact with drug users should be well knowledgeable about diagnosing and ruling out chronic respiratory diseases. More research is required to determine whether the diagnosis and treatment of respiratory illness in opium users are adequate. This may enable the detection of the proportion of opium users who also have undetected respiratory conditions, which may then be used to assess the effectiveness of existing therapy and bring about a change in policy.

## Conclusions

When controlling for tobacco and opium use, these population demonstrated greater prevalence of respiratory illnesses (asthma and COPD) than matched non-user controls. The findings of this study may have an impact on current harm-reduction strategies as well as encourage medical professionals to utilize a high threshold for identifying respiratory diseases among tobacco and opium users. Future studies should examine the potential causes of this connection as well as the prevalence of misdiagnosed and poorly treated respiratory diseases among this at-risk population.

## Data Availability

All data regarding this case has been reported in the manuscript. Please contact the corresponding author if you are interested in any further information.
